# Comparative Proteomics Analysis Reveals New Features of the Oxidative Stress Response in the Polyextremophilic Bacterium *Deinococcus radiodurans*

**DOI:** 10.3390/microorganisms8030451

**Published:** 2020-03-23

**Authors:** Lihua Gao, Zhengfu Zhou, Xiaonan Chen, Wei Zhang, Min Lin, Ming Chen

**Affiliations:** Biotechnology Research Institute, Chinese Academy of Agricultural Sciences, Beijing 100081, China; gaolihua89@hotmail.com (L.G.); zhouzhengfu@caas.cn (Z.Z.); 15933529573@163.com (X.C.); zhangwei01@caas.cn (W.Z.); linmin57@vip.163.com (M.L.)

**Keywords:** *Deinococcus radiodurans*, oxidative stress response, proteome, antioxidant system

## Abstract

*Deinococcus radiodurans* is known for its extreme resistance to ionizing radiation, oxidative stress, and other DNA-damaging agents. The robustness of this bacterium primarily originates from its strong oxidative resistance mechanisms. Hundreds of genes have been demonstrated to contribute to oxidative resistance in *D. radiodurans*; however, the antioxidant mechanisms have not been fully characterized. In this study, comparative proteomics analysis of *D. radiodurans* grown under normal and oxidative stress conditions was conducted using label-free quantitative proteomics. The abundances of 852 of 1700 proteins were found to significantly differ between the two groups. These differential proteins are mainly associated with translation, DNA repair and recombination, response to stresses, transcription, and cell wall organization. Highly upregulated expression was observed for ribosomal proteins such as RplB, Rpsl, RpsR, DNA damage response proteins (DdrA, DdrB), DNA repair proteins (RecN, RecA), and transcriptional regulators (members of TetR, AsnC, and GntR families, DdrI). The functional analysis of proteins in response to oxidative stress is discussed in detail. This study reveals the global protein expression profile of *D. radiodurans* in response to oxidative stress and provides new insights into the regulatory mechanism of oxidative resistance in *D. radiodurans*.

## 1. Introduction

Oxidative stress is ubiquitous and is induced by three main reactive oxygen species (ROS): hydroxyl radical (·OH), hydrogen peroxide (H_2_O_2_), and superoxide (O_2_^−^) [1], which are produced either during metabolic processes or following exposure to physical and chemical agents such as ionizing radiation [1], desiccation [2], ultraviolet radiation [3], and mitomycin [4]. Oxidative stress induced by ROS is harmful to all organisms. In humans, the oxidative modification of cellular macromolecules by ROS is the basis of various degenerative diseases, cancer, and aging [5,6].

The extremophilic bacterium *Deinococcus radiodurans* is well known for its extreme resistance to various stresses including ionizing radiation (IR), oxidative stress, and desiccation [6,7]; however, the mechanisms responsible for the extreme resistance of this robust bacterium remain unclear. At high doses of IR, *D. radiodurans* can rapidly reassemble DNA fragments into complete chromosomes [8,9]. Over the past few years, an effective DNA repair system was considered to be the decisive factor in ionizing radiation resistance of *D. radiodurans* [6]; however, compared to radiation-sensitive bacteria such as *Escherichia Coli*, no specific DNA damage repair protein has been identified in *D. radiodurans* [10,11]. There was no evidence of increased activity of DNA damage repair proteins (RecA and PolA) in *D. radiodurans*. In addition, a large number of studies have suggested that while the genome of *D. radiodurans* is as sensitive to IR as other organisms [12] its proteome is more resistant to oxidative damage than other radiation-sensitive organisms [9]. These studies indicate that it is the level of protein damage, rather than the level of DNA damage, that determines the bacteria’s resistance to ionizing radiation. For *D. radiodurans*, its potent antioxidant system is the determinant of its strong resistance to various stresses, which protects the proteome from oxidative damage, allowing the DNA repair system and metabolic system to function normally, thus restoring the cell to its normal physiological state. The antioxidant system of *D. radiodurans* includes not only the ROS-scavenging enzymes (such as catalase and superoxide dismutase) but also many non-enzymatic antioxidants (such as manganese complexes and carotenoids) as well as transcriptional regulators (PprM, IrrE, DdrA), and sRNA [13]. In addition to an effective DNA repair system, *D. radiodurans* also employs other strategies to prevent oxidative stress, including (1) prevention of the formation of endogenous ROS; (2) activation of the antioxidant defense system; (3) selective protection of the oxidative damage of certain proteins; and (4) removal and degradation of damaged macromolecules [7,14]. 

Proteins are the functional entities of organisms that affect the cell phenotype and stress response. In bacteria, the correlation between mRNA and protein abundance is not very strong [15]. The expression level of proteins is controlled by transcriptional regulation, transcript stability, and translational regulation [16]. The function and activity of proteins are usually regulated by post-translational modification, protein–protein interactions, and proteolysis [17]. The abundance and functional activity of protein also could not be determined by the analysis of transcript abundance and gene knockouts [17]. Therefore, it is necessary to conduct a systematic and comprehensive proteomic analysis to reveal the mechanism that accounts for the extreme resistance of *D. radiodurans* to oxidative stress. The latest advances in omics techniques provide a powerful tool for elucidating the molecular mechanisms of extreme resistance in this bacterium. Global protein analysis of *D. radiodurans* in response to ionizing radiation and desiccation stress has been reported, but the protein expression profiles of *D. radiodurans* under oxidative stress have not previously been studied. 

In this study, we utilized label-free quantitative proteomics analysis to examine the changes in the proteome of *D. radiodurans* in response to oxidative stress. The biological activities of differential proteins were analyzed by Gene Ontology (GO) and Kyoto Encyclopedia of Genes and Genomes (KEGG). These results indicate that the proteins involved in ribosome metabolism, translation, DNA repair and recombination, transcriptional regulation, stress response, cell wall organization, membrane composition, and transport are highly expressed under conditions of oxidative stress. This work integrates the information on proteome changes observed in this study, the expression levels of some oxidative-related genes, and the phenotypic outcomes of several gene knockout mutations shown in previous studies [5,7,14] to reveal some new features underlying oxidative stress resistance in *D. radiodurans*.

## 2. Materials and Methods 

### 2.1. Strain and Culture Conditions

*D. radiodurans* R1 ATCC BAA-816 was purchased from the China General Microbiological Culture Collection Center (CGMCC, Beijing, China) and cultured in TGY (Tryptone Glucose Yeast) broth (1% tryptone, 0.1% glucose, and 0.5% yeast extract) at 30 °C with shaking at 200 rpm or on TGY plates. 

### 2.2. Cell Survival under Oxidative Stress 

The susceptibility of *D. radiodurans* to H_2_O_2_ was determined as in previous research [18]. Strains were cultured overnight in TGY broth at 30 °C and were grown in fresh TGY broth up to an optical density at 600 nm (OD_600_) of 0.8. Then, the bacterial cells were treated with 80 mM H_2_O_2_ for 0, 10, 20, 30, and 50 min. After H_2_O_2_ shock, different serial 10-fold dilutions of these cells (10 μL) were spotted on TGY plates and incubated at 30 °C for 3 day. The survival rate was expressed as the percentage of the number of colonies in the treated samples compared with that of the untreated sample, which was used as a control [19]. A total of 100 μL of dilutions (10^−3^) was plated onto the TGY plate to calculate the number of colony-forming units (CFU). These experiments included three independent biological repeats.

### 2.3. Transmission Electron Microscope (TEM) 

For TEM analysis, the strains grown under normal growth conditions and oxidative stress conditions were washed twice with 1× PBS buffer and were collected by centrifugation. The cells were incubated overnight at 4 °C with 2.5% glutaraldehyde, centrifuged at 4000× *g* for 5 min, and then embedded in 2% agarose. The slices were stained with uranyl acetate for 15 min and observed on a Hitachi H-7650 transmission electron microscope.

### 2.4. Protein Extraction and Digestion

Strains grown to OD_600_ = 0.8 were used as samples. The samples without H_2_O_2_-shock were used as control samples (DC), and the samples treated with 80 mM H_2_O_2_ for 30 min were used as treated samples (DH). Each group of samples included three replicates. Bacterial total protein was extracted by ultrasonic crushing. In brief, the bacteria were ground into powder with liquid nitrogen, 100 μL lysozyme was added to the powder, and the solution was incubated at 37 °C for 30 min. Afterwards, 500 μL of urea (8M) was added, and ultrasonic crushing was performed with 30% ultrasonic power. Total protein was obtained by centrifugation at 14,000× *g* for 30 min. Protein concentrations were determined by the Bradford method [20] using bovine serum protein as the standard.

### 2.5. LC–MS/MS (Liquid Chromatography-Mass Spectrometry/Mass Spectrometry) Analysis by Q-Exactive and Sequence Database Searching

LC–MS/MS analysis was carried out according to the previous method [21]. The peptides were separated by a 90 min gradient elution at a flow rate of 350 nL/min with a Thermo Scientic EASY-nLC 1000 System (Nano HPLC), which was directly connected to a Thermo Scientific mass spectrometer (Q-Exactive). The pre-column was an Acclaim PepMap 100 column (2 cm × 100 μM, C18, 5 μM), and the chromatographic column was an EASY-Spray column (12 cm × 75 μm, C18, 3 μm). Mobile phase A consisted of 100% ultrapure and 0.1% formic acid, and mobile phase B consisted of 100% acetonitrile and 0.1% formic acid. The current velocity of the loading pump was 350 nL/min for 15 min. The mass spectrum parameters were as follows: (1) for the ion source parameters, the spray voltage was 2.1 kv, the capillary temperature was 250 °C, and the ion source was EASY-Spray source; (2) for the full-scan mass spectrum, the resolution was 70000 FWHM, the full-scan AGC target was 1 × 10^6^ the full-scan maximum IT was 60 ms, and the scan range was 350–1800 *m/z*; and (3) for dd-MS2, the resolution was 17500 FWHM, the AGC target was 5 × 10^6^; the maximum IT was 70ms; and the intensity threshold was 5 × 10^3^.

The mass spectrometry analysis of LC–MS/MS was performed using a Thermo Q-Exactive mass spectrometer, and the MS/MS spectra from each LC–MS/MS run were searched against *D. radiodurans*. fasta from UniProt using MaxQuant software (Version 1.5.2.8, Matthias Mann, Martinsried, Germany). The search criteria were as follows: full tryptic specificity was required; C-carboxyamidomethylation was set as the fixed modification; M oxidation and N-terminal were set as the variable modifications; the precursor ion mass tolerance was set at 15 ppm for all MS; the fragment ion mass tolerance was set at 20 mmu; and two missed cleavages were allowed.

### 2.6. GO and KEGG Enrichment Analysis

The GO and KEGG enrichment analyses were performed by Blast2go (version 5.2, Biobam, Valencia, https://www.blast2go.com/) and DAVID (version 6.8, Laboratory of Human Retrovirology and Immunoinformatics, Maryland, https://david.ncifcrf.gov), respectively. GO terms and KEGG pathways with corrected *p*-values of less than 0.05 were considered to be significantly enriched for differentially expression proteins (DEPs).

### 2.7. Protein–Protein Interaction Prediction

All differential proteins were input into the online software STRING (version 11.0, https://string-db.org/) for the prediction of protein–protein interactions, and the proteins that did not have close relationships were removed. The remaining proteins were input into Cytoscape software for mapping, and the node degree of each protein was counted.

### 2.8. RNA Isolation and Quantitative Real-Time PCR (qRT-PCR)

Total RNA isolation and qRT-PCR were performed exactly as described earlier [22]. Total RNA was isolated with TRIzol reagent and a PureLink RNA Mini kit (Invitrogen, Thermo Fisher, MA, USA). RNA (1 μg) was reverse-transcribed to cDNA using a PrimeScript^TM^ RT reagent kit with gDNA Eraser (TaKaRa Bio, Takara, Japan). qRT-PCR assays were performed with cDNA (1 μL) obtained from three independent cultures on a 7500 Fast Real-Time PCR System. The primer details are listed in Supplementary Table S1. The amplification conditions of the qRT-PCR assays were as follows: 95 °C for 5 min, followed by 40 cycles of 95 °C for 30 s, 60 °C for 1 min, and 72 °C for 30 s and then a dissociation curve analysis. The 16S rRNA gene was used as the endogenous reference gene to normalize the expression of target genes in each cDNA template [23]. The relative expression level of target genes was calculated by the comparative threshold cycle (2^−∆∆*C*t^) method. The qRT-PCR assays were performed using total RNA preparations obtained from three independent cultures (three biological replicates).

## 3. Results

### 3.1. Survival of D. radiodurans under Oxidative Stress

**Figure 1:**
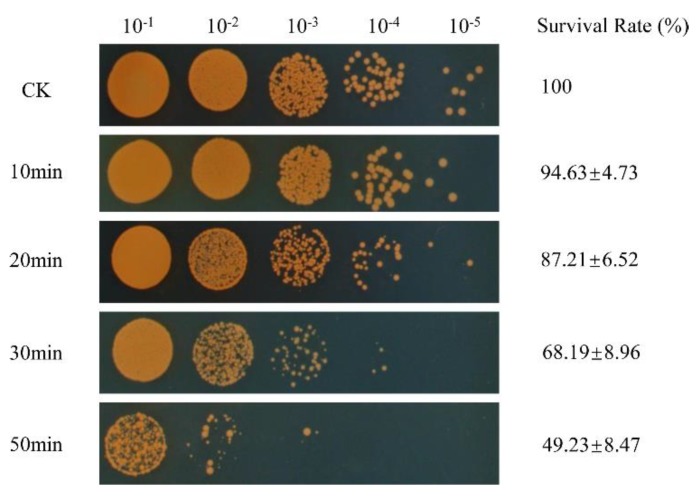
Survival phenotype plate assay of *Deinococcus radiodurans* exposed to 80 mM of H_2_O_2_ for different shock times (0, 10, 20, 30, 50 min). CK: untreated sample control. The survival rate is expressed as the percentage of the number of colonies in the H_2_O_2_-treated samples compared with that in the untreated sample control. The survival rates of different samples were obtained from 10^−3^ dilution with three independent repeats.

To investigate the adaptive mechanism of *D. radiodurans* to oxidative stress, we used 80 mM of H_2_O_2_ to treat *D. radiodurans* wild-type strains with a series of treatment times (0, 10, 20, 30, 50 min) to determine the optimal proteome measurement conditions. We found that the survival rate of *D. radiodurans* gradually decreased with the extension of the H_2_O_2_-shock time. When the H_2_O_2_-shock time reached 30 min, the survival rate of the treated samples dropped to 68% compared to that of the untreated samples (CK) (Figure 1). At this time, the expression levels of a large number of proteins had changed, so this condition was selected for quantitative proteomics analysis of *D. radiodurans* under oxidative stress.

**Figure 2:**
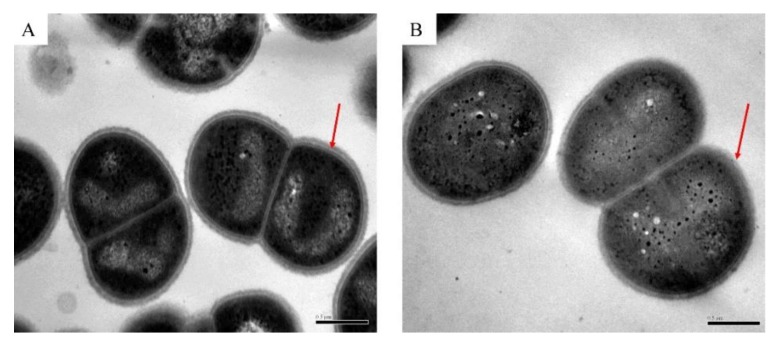
Transmission electron microscopy (TEM) images of *D. radiodurans* cells (**A**) under normal growth conditions and (**B**) following exposure to 80 mM H_2_O_2_ for 30 min. Bar: 500 nm. The red arrow points to the cell wall and cell membrane.

Since oxidative stress may affect the integrity of the cell wall and cell membrane of *D. radiodurans*, we used transmission electron microscopy (TEM) to observe the morphology of *D. radiodurans* cells under normal growth (DC) and oxidative stress conditions at a concentration of 80 mM H_2_O_2_ for 30 min (DH). As shown in Figure 2A,B, the cell ultrastructure of *D. radiodurans* with H_2_O_2_ treatment (DH) was different from that of *D. radiodurans* without H_2_O_2_ treatment (DC). DH exhibited some visible damage in some parts of the cell membrane while the bilayer of the cell membrane of DC was intact, which suggests that external oxidative stress first destroys the bacterial envelope and then enters into the cell to damage other macromolecules. The bacterial envelope provides the first line of defense against external stresses.

### 3.2. Global Analysis of the Differentially Expressed Proteins (DEPs) in DC and DH

**Figure 3:**
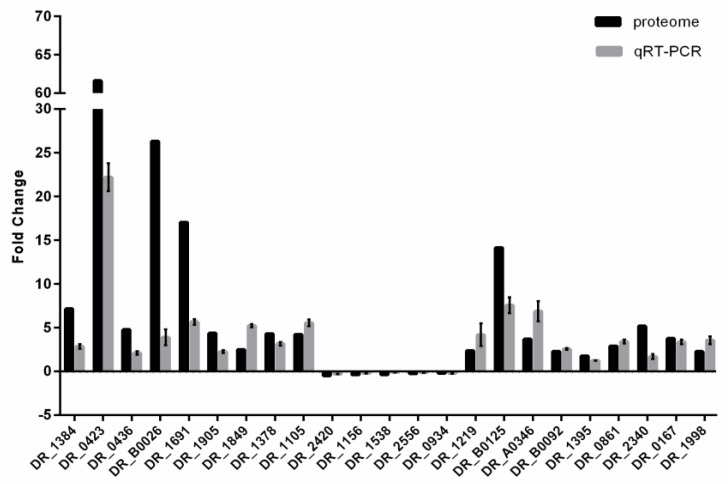
Comparison of the fold change in expression levels of differential proteins detected by Proteomic and Quantitative Real-Time PCR (qRT-PCR). The qRT-PCR data are presented as the mean ± SD values of triplicate reactions for each gene transcript. The selected proteins belong to different functional categories; the annotation of each gene is detailed in Supplementary Table S1.

In this study, 1470 and 1700 proteins were respectively identified in DC and DH using LC–MS/MS. Only proteins that were detected in both samples could be subject to differential analysis. A total of 852 proteins were found to be differentially expressed by the one-way ANOVA (analysis of variance) analysis (fold change > 1.5 and *p* value < 0.05). Among these differentially expressed proteins (DEPs), 711 proteins were upregulated in DH, whereas 141 proteins were downregulated in DH (Supplementary Figure S1). This indicates that the expression of most DEPs under oxidative stress conditions is upregulated. Several DEPs representing different functional categories were selected for quantitative real-time PCR (qRT-PCR) analysis. With the exception of *DR_0423* (DNA damage response protein A, DdrA), *DR_B0026* (sigma B regulator, RsbT), *DR_1691* (heat shock protein-related protein), and *DR_B0125* (iron ABC transporter), the expression profiles of most genes showed similar tendencies to those detected by proteomics assay, indicating the good quality of the sequencing data (Figure 3). As for these four genes, the significant difference in fold changes in expression levels detected by proteome analysis and qRT-PCR may have been caused by regulation at the post-transcriptional and translational levels.

### 3.3. Gene Ontology (GO) and Kyoto Encyclopedia of Gene and Genome (KEGG) Analyses of the DEPs

**Figure 4:**
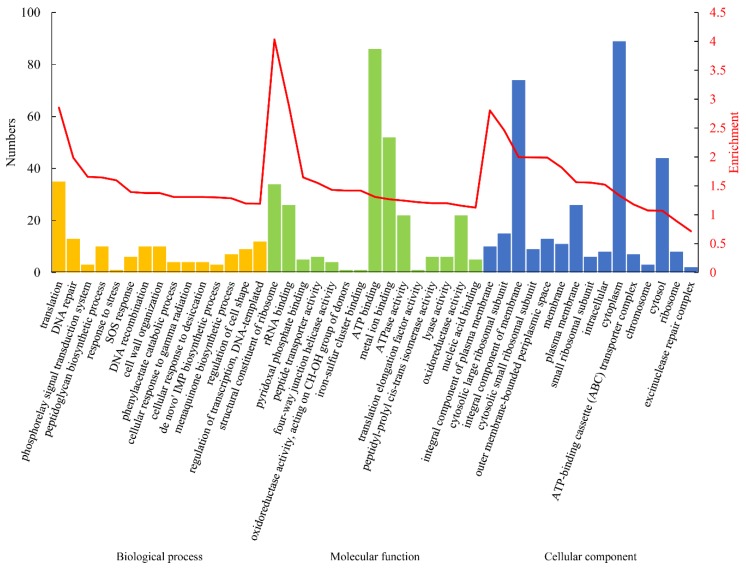
Gene ontology (GO) enrichment analysis of differentially expressed proteins between DC (control samples) and DH (treated samples). The vertical histogram represents the number of differential proteins enriched in each GO term; the red line represents the enrichment degree (−log_10_ *p* value) of each GO term. The 45 most enriched GO terms are shown.

**Figure 5:**
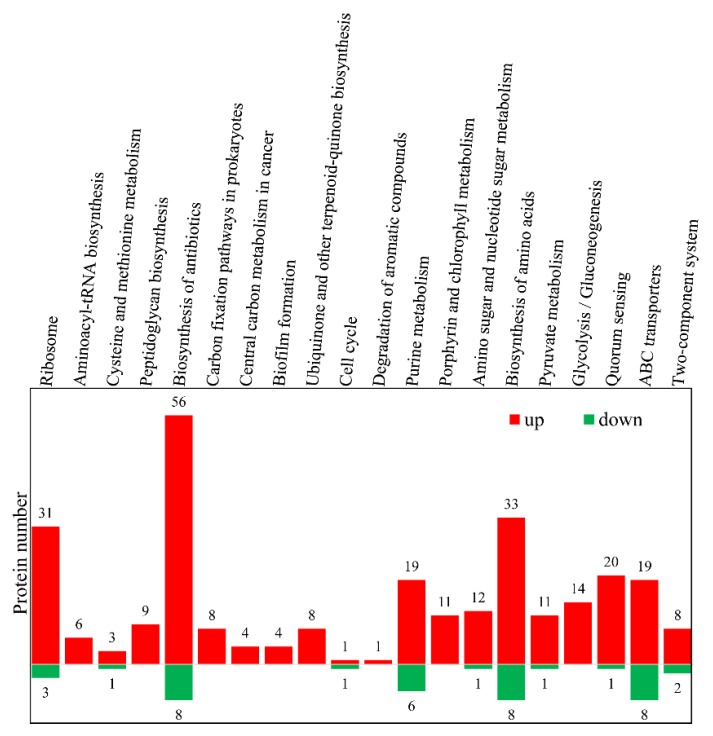
Top 20 Kyoto Encyclopedia of Genes and Genomes (KEGG) biological pathway classification histograms for differential proteins in DH versus DC. The x-axis represents the enriched KEGG pathway, and the y-axis represents the number of proteins that were significantly upregulated or downregulated expressed in DH versus DC.

To decipher the biological relevance and understand the functional characteristics of the DEPs, Gene Ontology (GO) enrichment analysis was performed, and the results (Figure 4) revealed significant enrichment in biological processes and molecular functions relevant to translation, DNA repair and recombination, response to stress, SOS response, cellular response to gamma radiation and desiccation, and metabolism. These DEPs are predominantly components of the membrane (integral component of plasma membrane, integral component of membrane, plasma membrane) and the cytosolic large/small ribosomal subunit, and most of them have binding abilities (rRNA binding, pyridoxal phosphate binding, iron–sulfur cluster binding, ATP binding, metal ion binding) and oxidoreductase activity. These data imply that a large number of proteins with different functions are differentially expressed as a result of oxidative stress in *D. radiodurans*. To obtain an overview of the biological functions of these DEPs during the oxidative stress response, these differential proteins were annotated with KEGG functional groups; the distributions in each category are shown in Supplementary Figure S2. The proteins involved in biological pathways related to the biosynthesis of antibiotics, biosynthesis of amino acids, ribosomes, purine metabolism, quorum sensing, and ABC transporter were significantly induced by oxidative stress (Figure 5). These results indicate that *D. radiodurans* utilizes numerous biological processes and pathways in response to oxidative stress.

According to the COG (Clusters of Orthologous Groups) functional classification, the DEPs were further classified into three major categories: metabolism, information storage and processing, and cellular processes and signaling. Among them, translation, ribosomal structure, and biogenesis accounted for a maximum of 12.09%; amino acid transport and metabolism accounted for 11.62%; and cell wall/membrane/envelope biogenesis accounted for 7.38% (Supplementary Figure S3). The COG analysis results are consistent with the previous GO and KEGG enrichment analyses.

### 3.4. Protein–Protein Interaction (PPI) Network of DEPs

**Figure 6:**
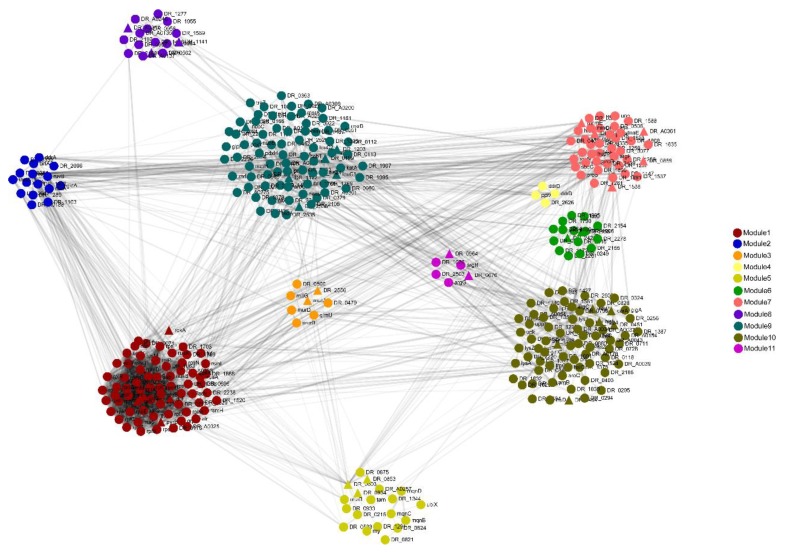
The protein–protein interaction (PPI) network of oxidative stress response proteins in *D. radiodurans* revealed by functional protein association network (STRING) analysis. A total of 336 differential proteins are shown in the PPI network, with 11 modules indicated in different colors. Module 1: translation; Module 2: DNA repair; Module 3: cell wall organization; Module 4: cellular response to gamma radiation; Module 5: catalytic activity; Module 6: regulation of transcription; Module 7: ATP/GTP/DNA/RNA/protein binding; Module 8: membrane and transport; Module 9: oxidation–reduction process; Module 10: unclassified; Module 11: protein folding and degradation.

The oxidative stress resistance of *D. radiodurans* is controlled by many proteins. These proteins constitute a complex regulatory network containing multiple biological process, the realization of which requires the cooperation of multiple proteins. The characterization of the protein–protein interaction network provides important evidence for revealing the regulatory mechanism of oxidative resistance in *D. radiodurans*. A total of 852 DEPs were analyzed for protein interactions using the online software STRING, of which 336 proteins were described in the PPI network. Eleven modules formed tightly connected clusters, and the thickness of the lines in the network indicate the strength of the associations (Figure 6). The node degree of each protein is shown in Supplementary Table S2. Module 1 contains proteins mainly involved in translation. The proteins with a high node degree were SecY, RpsE, RpsC, RplM, RpsK, RplB, and RplR. Module 2 includes the proteins mainly involved in DNA repair; among them, RecA shows the highest node degree. Modules 3–8 contain proteins mainly involved in cell wall organization, cellular response to gamma radiation, catalytic activity, regulation of transcription, binding activity, and membrane transport. Module 9 includes those proteins mainly involved in the oxidation–reduction process, among them, the proteins with high node degrees are DR0379, DRA0011, IrrE, GltA, DR2242, and FabZ. Module 11 contains proteins mainly involved in protein folding and degradation. These data indicate that the DEPs produced in response to oxidative stress are mainly involved in translation and oxidation–reduction processes. 

### 3.5. Differential Proteins between DC and DH Enriched in Antioxidant Activity

The previous GO and KEGG enrichment analyses in our research have suggested that most DEPs induced in response to oxidative stress belong to the functional categories that are closely related to the oxidative stress response, such as translation, transcription, DNA repair, stress responses, and SOS response. Here, we focus on the biological characteristics and functional analysis of several major oxidative stress response proteins.

#### 3.5.1. Catalase Detoxification of H_2_O_2_

Catalase is a metalloenzyme that converts H_2_O_2_ to water and O_2_, thus protecting organisms from oxidative damage caused by H_2_O_2_ [24]. Catalases are divided into three families, namely monofunctional heme catalases (KatEs), bifunctional heme catalase peroxidases (KatGs), and manganese catalases (MnCats) [25,26]. There are three catalases in *D. radiodurans*: DR1998 (KatE1), DRA0259 (KatE2), and DR_A0146. The first two are constitutively expressed under normal conditions [27,28]. DR_A0146 is a eukaryotic-type catalase. KatE1 is the most important catalase in *D. radiodurans*; it has a higher activity of 68,800 U/mg and is a more efficient scavenger of H_2_O_2_ than KatE2 and DRA0146 [28,29]. The proteome data showed that DR1998 was upregulated by 2.29-fold under conditions of oxidative stress. These results suggest that DR1998 plays an important role in the oxidative resistance of *D. radiodurans*.

#### 3.5.2. DNA-Binding Proteins during the Stationary Phase (Dps)

Cellular iron homeostasis is very important in the life processes of all living organisms. Dps, as a ferritin protein, plays an important role in the detoxification of ROS, iron scavenging, and the mechanical protection of DNA [30], and is distributed widely in the bacterial kingdom. The Dps of *E. coli* protects its DNA from various types of damage by promoting compaction of its DNA [31]. In addition to promoting nucleoid compaction, Dps protects cells from oxidative damage by isolating Fe^2+^ and decomposing H_2_O_2_ [32,33]. *D. radiodurans* encodes two Dps, Dps1 (DR_2263), and Dps2 (DR_B0092). Dps1 has a high affinity for DNA binding and is located in the nucleoid of *D. radiodurans*, while Dps2 has a low binding affinity with DNA [34,35]. Dps1 is constitutionally expressed at different growth stages in *D. radiodurans* under normal growth conditions, while Dps2 is significantly upregulated under conditions of oxidative stress [34,36], which is consistent with the proteome data collected in this study, where Dps1 was shown not to induced by oxidative stress, while Dps2 expression was increased by 2.3-fold under these conditions. 

#### 3.5.3. Proteins Involved in Carotenoid Synthesis

Carotenoids are widespread natural pigments that play an important role in cellular oxidative protection. In phototrophic bacteria, carotenoids play a photoprotective role against the destruction of free radicals generated by light and oxygen [37], while in non-phototrophic bacteria, carotenoids protect cells from oxidative damage as ROS scavengers. Most extremophiles contain carotenoids [38]. The major carotenoid in *D. radiodurans* is deinoxanthin, which gives the bacterium a distinctive orange-red color and shows a higher scavenging activity on H_2_O_2_ [39,40]. A pigmentless mutant of *D. radiodurans* exhibited greater sensitivity to H_2_O_2_ treatment than the wild type [41]. *D. radiodurans* contains a number of proteins involved in carotenoid synthesis, such as geranylgeranyl diphosphate synthase (CrtE, DR1395) and phytoene desaturase (CrtI, DR0861). The proteome data in our study showed that the expression level of DR1395 and DR0861 increased by 1.79- and 2.91-fold, respectively, under conditions of oxidative stress. These results suggest that the upregulated expression of these proteins under conditions of oxidative stress contributes to an increase in the content of carotenoids in *D. radiodurans*, thus improving the oxidative resistance of *D. radiodurans*.

#### 3.5.4. Mn^2+^/Fe^2+^ Homeostasis

Metal ions are important enzyme cofactors of proteins involved in DNA synthesis and repair, electron transport, and ROS scavenging [1]. The intracellular Mn^2+^/Fe^2+^ ratios are 70- to 300-fold higher in *Deinococcus* species as compared to those in radiation-sensitive bacteria [42]. It has been reported that the high Mn^2+^/Fe^2+^ ratio is closely associated with the strong resistance of bacteria to radiation and desiccation as well as the low levels of protein oxidation [9]. In *D. radiodurans*, the proteins involved in Fe^2+^ transport include an ABC-type hemin transporter (DR_B0016), an ABC-type Fe^3+^ siderophore transporter (DR_B0017), and two Fe^2+^ transporters (DR_1219, DR_1220) [1]. The proteome data in our study show that the expression level of DR_1219 was upregulated by 3-fold under conditions of oxidative stress. DR_1219 (ferrous iron transport protein B) and DR_B0125 (Fe^3+^ dicitrate-binding periplasmic protein) are involved in ferrous and ferric iron uptake, respectively. DR_B0125 was upregulated 14.15-fold under the conditions of oxidative stress in this study. However, the action mechanism of these iron transporters is still poorly understood, and in-depth study of these important proteins is necessary to reveal the mechanism responsible for iron transport in *D. radiodurans*.

#### 3.5.5. The Cyclic AMP (Adenosine MonoPhosphate) Receptor Protein DdrI

Cyclic AMP receptor proteins, as global transcription regulators, regulate various metabolic pathways and stress responses, including the oxidative stress response. The genome of *D. radiodurans* encodes four cyclic AMP receptor proteins. Among them, DR_0997 (DdrI) levels increased by 6.75-fold under conditions of oxidative stress. The *DdrI* deletion mutant showed significant sensitivity to heat shock, oxidative stress, and DNA damage, indicating that DdrI plays an important role in protecting *D. radiodurans* from these stresses [43,44]. Moreover, catalase activity also decreased in the *ddrI* deletion mutant under normal growth conditions and oxidative stress conditions [43]. The results of electrophoretic gel mobility shift assays showed that the transcript levels of eight genes—DR_1998, DR_0349, DR_1974, DR_1506, DR_2531, DR_1819, DR_A0346, and DR_1447—were directly regulated by DdrI [43]. Among them, the expression levels of DR_A0346 (PprA) and DR_1447 (RecN) increased by 3.69- and 1.99-fold, respectively. In short, DdrI plays multiple regulatory roles in many biological processes such as DNA repair and antioxidant metabolism. 

#### 3.5.6. Radiation Response Metalloprotease IrrE

IrrE (also referred to as PprI) is considered to be a broad-spectrum transcription factor that regulates many stress responses, including against oxidative stress, IR, and desiccation. It is only found in Deinococcaceae and Thermaceae bacteria [45,46,47]. The proteomic data in our study showed that the expression of PprI was upregulated 3.77-fold in response to oxidative stress. The *PprI* mutant exhibited high sensitivity to IR and decreased catalase activities in response to IR [45]. Furthermore, the overexpression of *IrrE* in *E. coli* was shown to significantly increase the catalase activities as well as radiation, osmotic, and salt tolerance [48,49,50,51]. These results suggest that PprI not only participates in various stress responses as a global regulator in *D. radiodurans*, but also increases the stress tolerance of other organisms.

## 4. Discussion

It has been suggested that the proteome of *D. radiodurans* is well-protected by the antioxidant system after exposure to IR [7,14]. The undamaged proteins perform their function of repairing injured cellular macromolecules (DNA, lipids, and proteins), ultimately restoring the cell to its normal physiological state. In this study, we used a label-free proteomics method to investigate the mechanism of adaptation to oxidative stress in *D. radiodurans*. After oxidative stress treatment at a concentration of 80 mM H_2_O_2_ for 30 min, a large number of proteins were differentially expressed to allow *D. radiodurans* to adapt to the external oxidative stress. The cell wall is crucial for cellular function, especially for ensuring normal physiological activities occur in the cell because it separates the internal environment from the external environment of the cell. The TEM results show that the integrity of cell walls and cell membranes of *D. radiodurans* was damaged by oxidative stress, which is consistent with the results of the GO analysis that showed that many differential proteins were concentrated in “cell wall organization”, “integral component of plasma membrane”, and “membrane”. Previous studies showed that heat stress also can damage the integrity of the cell wall in *E. coli* [40,41] and *D. radiodurans* [22]. These results indicate that the external stresses first destroy the cell walls of bacteria, breaking the cell’s first line of defense against external pressure, and then affect the physiological activities in cells. 

The differential proteins observed in this study in response to oxidative stress were mainly enriched in the following biological processes: translation, DNA repair and recombination, SOS response, cellular response to desiccation and gamma radiation, cell wall organization, and transcription. Previously, a proteome analysis completed by Aman et al. [52] indicated that proteins induced by desiccation and gamma radiation in *D. radiodurans* were significantly enriched in biological processes including DNA repair; cellular responses to reactive oxygen, gamma radiation, and desiccation; and DNA replication. The transcriptomic analysis of *D. radiodurans* under heat stress as reported by Xue et al. [22] revealed that heat stress-induced genes were mainly involved in the response to stress and stimulus, proteolysis, oxidation–reduction processes, DNA repair, and other biological processes. All of these studies revealed a convergence and similarity in oxidative stress, desiccation, gamma radiation, and heat stress response pathways in *D. radiodurans*, suggesting that some biological processes, including DNA repair and response to stresses (oxidative stress, desiccation, gamma), are very critical for *D. radiodurans* to respond to various stresses. The reasonable explanation for this conclusion may be that all stresses will produce ROS and induce oxidative damage to cells [5,53]. 

To the best of our knowledge, *D. radiodurans* has several certain proteins that are upregulated under conditions of oxidative stress, including KatE1, Dps, DdrI, IrrE, and other proteins with oxidative stress resistance. In our study, the expression levels of these proteins were also significantly upregulated during the oxidative stress response. KatE1, the major catalase, plays a critical role in the oxidative stress resistance of *D. radiodurans* by detoxifying H_2_O_2_ [28]. The Δ*katE1* mutant exhibited the greatest decrease in H_2_O_2_ resistance and the highest increase in intracellular ROS levels following H_2_O_2_ treatment among the three catalases (KatE1, KatE2, and DR_A0146) [28]. The KatE1-type catalase is common in the genus *Deinococcus* [28]. Dps proteins belong to the ferritin superfamily. Apart from promoting the compaction of the nucleoid, Dps proteins from *E. coli* also protect DNA from oxidative stress damage by binding DNA, chelating Fe^2+^, and reducing H_2_O_2_ to H_2_O [32,33]. Dps1 and Dps2 in *D. radiodurans* also carry out important functions in the homeostasis of intracellular metal (Fe and Mn) and DNA protection [34,35]. DdrI, as a global transcription regulator, plays an important role in regulating many stress responses. Previous research results indicated that the absence of *DdrI* significantly increases the sensitivity of *D. radiodurans* to oxidative stress, heat stress, and DNA damage [43,44]. IrrE is a novel regulatory protein considered to efficiently improve the DNA repair capability and the extreme radiation and oxidative stress resistance of *D. radiodurans* by regulating various pathways [45,46,47]. In addition to these classic oxidative stress response proteins, many new proteins with unknown functions were also found to be significantly upregulated following H_2_O_2_ treatment, such as DR_0644, DR_2446, DR_0399, and DR_0548. In the future, we will study the specific functions of these new proteins with a view to improving our understanding of the regulatory mechanisms of oxidative resistance in *D. radiodurans*. Interestingly, we found that the proteins with the greatest amount of upregulation under oxidative stress were generally ribosomal proteins. For example, rpsl, rpsR, and rpsT were upregulated 242-, 232-, and 196-fold, respectively. It is suggested that a large number of proteins need be resynthesized to replace those that are oxidatively damaged proteins when *D. radiodurans* is subjected to oxidative stress. Our findings are consistent with previous studies where transcription and translation elongation factors were found to be induced in response to IR to meet the need for rapid synthesis due to damaged proteins, particularly to replace those essential for the recovery process [27,54,55]. 

Taken together, the results of our study reveal a complex oxidative stress response in *D. radiodurans* that involves various biological process and metabolic pathways. These proteins, which are responsive to oxidative stress, are mainly associated with translation, DNA recombination and repair, response to stresses, transcription, and cell wall organization. Further functional analyses of these new oxidative stress-induced proteins will enhance our understanding of the regulatory mechanism underlying extreme resistance to oxidative stress in *D. radioduras*.

## Supplementary Materials

The following are available online at https://www.mdpi.com/2076-2607/8/3/451/s1, Figure S1: Differential expression levels between two groups of samples (DC and DH), Figure S2: KEGG biological pathway classification of differentially expressed proteins in *D. radiodurans* with and without oxidative stress, Figure S3: COG functional classification analysis of all the differential proteins in DH versus DC. Table S1: List of primers used for qRT-PCR in this study, Table S2: List of node degree of each protein in PPI.
