# Supplementary material for: Comparative Proteomics Analysis Reveals New Features of the Oxidative Stress Response in the Polyextremophilic Bacterium Deinococcus radiodurans
Source: Microorganisms. 2020 Mar 23;8(3):451. doi: 10.3390/microorganisms8030451 (PMC7143949; doi:10.3390/microorganisms8030451)
Supplement: Supplementary file 1 [file microorganisms-08-00451-s001.pdf]

Supplementary Material

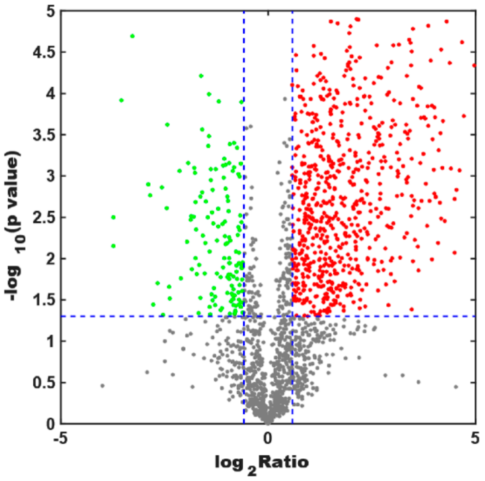

Supplementary Figure S1. Differential expression levels between two groups of samples (DC and DH). The x-axis represents the log_{10} ratio (fold change) of the expression level of every protein in each group. The y-axis represents the $-log_{10}$ (*p* value); the larger the y-axis value, the more significant the difference. Only proteins detected in both samples are shown. Each point represents a protein. The red points indicate significantly upregulated proteins in DH, and the green points indicate significantly downregulated proteins in DH. The criteria for screening differential proteins are ratio (fold change) > 1.5 and p value < 0.05.

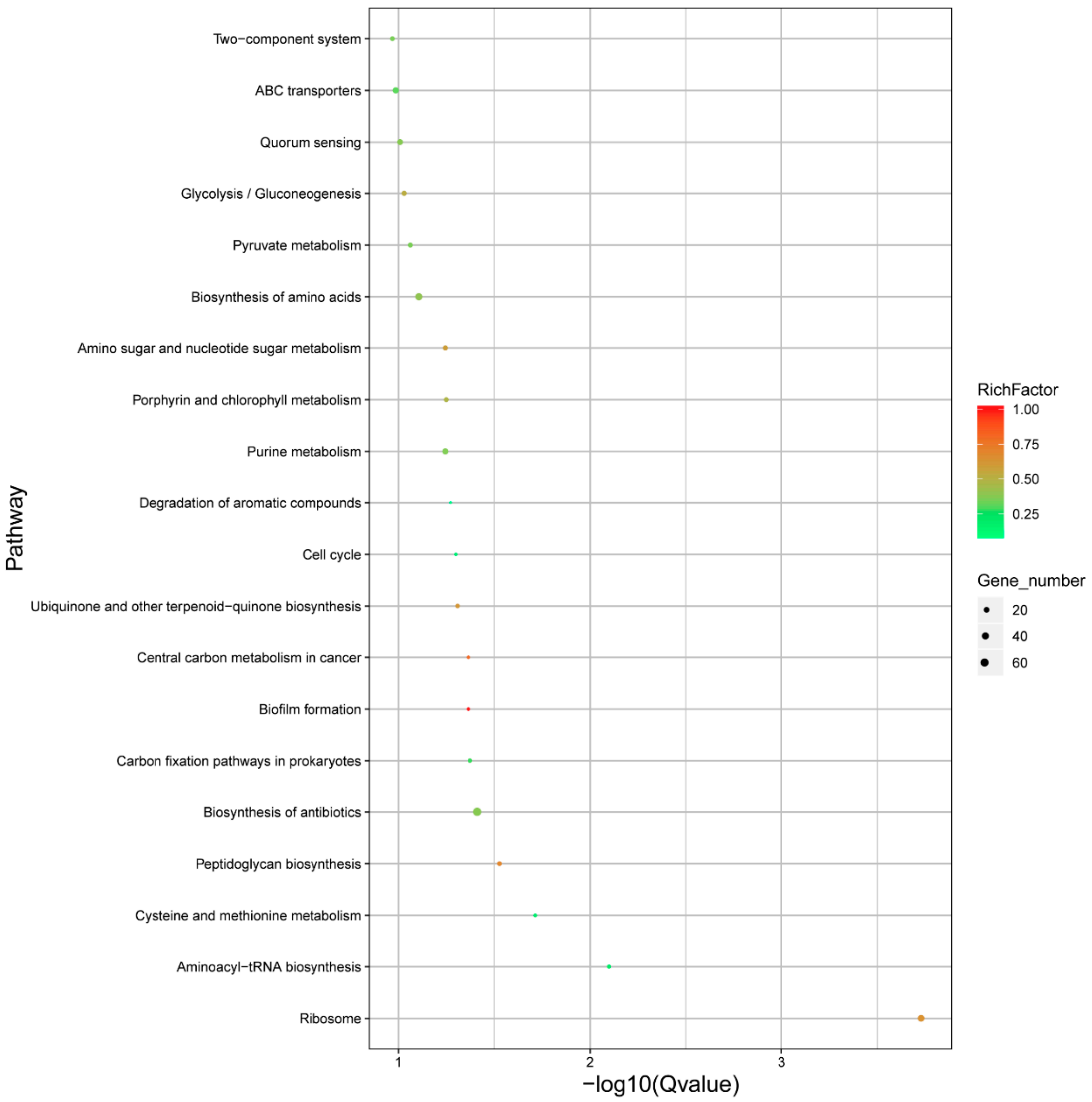

Supplementary Figure S2 KEGG biological pathway classification of differentially expressed proteins in *D. radiodurans* with and without oxidative stress. The 20 most enriched KEGG terms are shown.

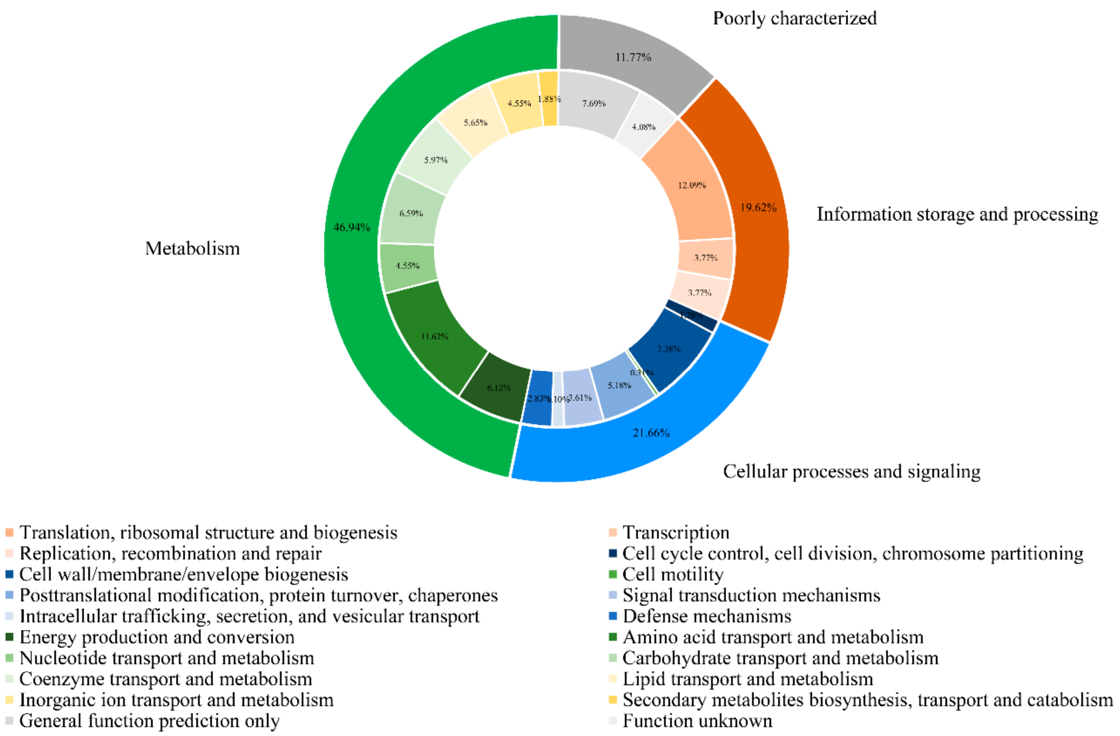

Supplementary Figure S3. COG functional classification analysis of all the differential proteins in DH versus DC.

Supplementary Table S1. List of primers used for qRT-PCR in this study

Gene name and annotation	Primers (5′ to 3′)
DR_1384	F: CTCTTCATCGAGCAGGGCTT
Transcriptional regulator, TetR family	R: CGAGTTCCGCGAAATACTGC
DR_0423	F: TTTCCCGCTCATACCGTGAG
DNA damage response protein A, DdrA	R: CCGGACACCACTTCCATCTC
DR_0436	F: TGCGTATCTACTTCACGGGC
Cytochrome B6	R: TGGTCGCCACCAGAATCATC
DR_B0026	F: CTCGGGGCTGAAAATGACCT
Sigma-B regulator, RsbT	R: CTCCGTCTCGATTCGCAGTT
DR_1691	F: GTCCGTGAGCTGGCCTTTC
Heat shock protein-related protein	R: AGGACTGGTTTCTGTGCTGG
DR_1905	F: ATTCGAGGAGCTGATGCAGG
Transcriptional regulator, GntR family	R: AGCAGGTGAAGCTGCATGTC
DR_1849	F: CCGCACAATAGCAGCATGAC
Peptide methionine sulfoxide reductase MsrA	R: GCTGATCTGGTTCGGGTCAA
DR_1378	F: GTACTGGGACCACGACGAAG
Peptide methionine sulfoxide reductase MsrB	R: GGGCGTAGCCGATCTTGTAG
DR_1105	F: TGCTGGCCTCTTCTTTTGCT
DNA repair protein RadA	R: TGGGCACTTCTTCCTCGAAC
DR_2420	F: CACCCGGACCATCGAGATTC

Response regulator	R: TCGGCCTTGATTTCCTCCAG
DR_1156	F: CTTCAGTGAACGCAACTCGC
Transcriptional regulator	R: ATCAACTGACTGGCCTCGAC
DR_1538	F: GGCAGATATTGCACGCAAGG
Osmotically inducible protein C	R: GTCGAGCGCCTTGATCTCAT
DR_2556	F: CGATGCCGTTCATCTTGCTC
Response regulator	R: CCAGCTGGTGACTTCCTTGT
DR_0934	F: CCCTGTCTACGTCATCTCGG
Transcriptional regulator, MerR family	R: GATTTCGGCCTCGAACTCCT
DR_1219	F：CTCGCCATGAGCTTTCTGGA
Ferrous iron transport protein B	R：CTCCCACTACGCGGAAAGAG
DR_B0125	F：ACTGAACACCAAAGCCACCA
Iron ABC transporter	R：TCGTTGCTGTTGCGGATACT
DR_A0346	F：GGCAGTATGGCAAGGGCTAA
DNA repair protein PprA	R：TTCTTGCAAGGACTGCGTGA
DR_B0092	F：GCAAAACACCTTGACCGAGC
DNA protection during starvation protein 2, dps2	R：CTGATGCCCTCGTAGTGGTC
DR_1395	F：CCTTCCAGATTCGGGACGAC
Geranylgeranyl diphosphate synthase	R：CCAGCGGTGAATCTCGTCAA
DR_0861	F：ACTCGGCTACACCCATTTCG
Phytoene dehydrogenase	R：AGCAGCGTCTCGAAGGAAAA
DR_2340	F：GCACCTGTGCGTTTATCGAC
RecA	R：GTTCCATGATTTCGAGCGCC
DR_0167	F：TAAGCTCCCATTCGTGACCG
Radiation response metalloprotease IrrE	R：CGCCGAGTTGATGAGGATGA
DR_1998	F：GGACTTCAGCGACGACAAGA
katA	R：TCGTAGTTCACACGCTGGTC
DR_r01	F: ATTCCTGGTGTAGCGGTG
16S ribosomal RNA	R: CATCGTTTAGGGTGTGGAC

Supplementary Table S2. List of node degree of each protein in *PPI*

Type	Protein name	Degree
Module1 Translation	SecY	50
	RpsE	50
	RpsC	49
	RplM	48
	RpsK	47
	RplB	47
	RplR	46
	Ffh	46
	RpsL	45

Module2 DNA repair	RecA	59
	RuvB	23
	UvrB	19
	CinA	16
	DR_1289	15
	DdrA	15
	RadA	12
	DR_0647	10
	GidA	8
	DR_A0188	6
Module3 Cell wall organization	MurD	14
	MurB	9
	DR_0479	8
	MurJ	7
	GlmU	6
Module4 Cellular response to gamma radiation	DdrB	10
	PprA	9
	DdrD	6
	DR_2626	1
Module5 Catalytic activity	DR_0603	43
	DR_0588	7
	MiaB	6
	MqnC	4
	MqnB	3
Module6 Regulation of transcription, DNA-templated	DR_1174	23
	LutC	4
	DR_1906	4
	DR_0997	4
	DR_2155	3
Module7 ATP/GTP/DNA/RNA/protein binding	DR_2444	21
	DR_0400	17
	DnaE	16
	ArgF	12
	AroB	10
	MnmE	10
	RecN	9
	SbcC	9
	HisS	9
	DR_1147	8
	DR_2198	7

Module8 Membrane and transport	DR_0562	5
	DR_1569	5
	DR_0958	5
	DR_0561	4
Module9 Oxidation-reduction process	DR_0379	24
	DR_A0011	22
	IrrE	19
	GltA	18
	DR_2242	16
	FabZ	15
	DR_1185	15
	DR_1115	15
	NuoC	14
	DR_1487	14
Module10 Unclassified	DR_0451	18
	Prs	18
	DR_A0034	14
	ArgB	9
	DR_A0054	8
Module11 Protein folding and degradation	ArgG	15
	ArgR	5
	DR_1070	4
	DR_0964	3
	DR_2503	2
